# In-to-Out Body Antenna-Independent Path Loss Model for Multilayered Tissues and Heterogeneous Medium

**DOI:** 10.3390/s150100408

**Published:** 2014-12-29

**Authors:** Divya Kurup, Günter Vermeeren, Emmeric Tanghe, Wout Joseph, Luc Martens

**Affiliations:** Ghent University-iMinds, Deptartment of Information Technology Gaston Crommenlaan 8 box 201, Ghent B-9050, Belgium; E-Mails: gunter.vermeeren@intec.ugent.be (G.V.); emmeric.tanghe@intec.ugent.be (E.T.); wout.joseph@intec.ugent.be (W.J.); Luc.Martens@intec.UGent.be (L.M.)

**Keywords:** path loss model, layered medium, antenna gain in conducting medium, WBAN, human body

## Abstract

In this paper, we investigate multilayered lossy and heterogeneous media for wireless body area networks (WBAN) to develop a simple, fast and efficient analytical in-to-out body path loss (PL) model at 2.45 GHz and, thus, avoid time-consuming simulations. The PL model is an antenna-independent model and is validated with simulations in layered medium, as well as in a 3D human model using electromagnetic solvers.

## 1. Introduction

A wireless body area network (WBAN) consists of nodes that communicate wirelessly and are located in, on or outside the body of a person. To optimize the communication between the nodes placed within or outside the human body, a better understanding of propagation loss is required for the development of WBANs. This need arises, as the human body is a lossy medium, which considerably attenuates the electromagnetic waves traveling from the transmitter (Tx) to the receiver (Rx).

The novelty of this research paper is the development of a simplified generic path loss (PL) model, which describes the signal attenuation between a Tx and an Rx antenna as a function of the propagation distance and other parameters (for example, conductivity), applicable for heterogeneous human models for in-to-out body communication at 2.45 GHz and which is independent of the antenna characteristics [1,2]. We select the 2.45-GHz frequency corresponding to the industrial, scientific and medical (ISM) band, as this is one way to reduce the antenna size and to make it available for implantation. Another advantage is that the larger bandwidth allows for higher bitrates. Various antennas are manufactured for the same reason [3–5]. The voting by the FCC to include the 2.36–2.4 GHz spectrum in medical device radiocommunications (MedRadio) for body area networks is also a motivation for carrying out this study [6].

In our previous work, we have presented the path loss models for insulated dipole antenna in a homogeneous lossy medium to understand the influence of the conductivity and permittivity of the medium on the path loss [7]; further, we extended the path loss model to include the range of conductivities and permittivities of the human body [2]. However, these path loss models were antenna specific (dipole antenna). Thus, in [8], we developed a method to find the antenna gain in the conducting medium and developed a path loss model by using the calculated gain, thereby making the model independent of the antenna-type. However, these models were specific for homogeneous medium only. Further, the path loss models were developed for a heterogeneous medium in [9,10], but are applicable only for the virtual family model provided by SEMCAD-X. Thus, in this paper, we provide a generic path loss model for a heterogeneous medium.

A transmission channel includes both the antenna and channel properties that lie between the terminals of the two antennas. While designing communication systems for body-centric systems, PL models become more useful and widely applicable if the properties of the propagation channel can be extracted from the transmission channel [11]. Various PL models have been created for in-body and in-to-out body propagation by [1,2,7,12–14]. The main difference between these PL models and the simplified PL model developed in this paper is that all other PL models include antenna parameters, such as gain, and are only applicable for the considered antennas, thus limiting the general usability of the PL models. Antennas for in-body and on-body propagation have been designed [3,15] at 2.45 and 5.8 GHz, and a method to calculate the gain of these antennas in a conducting medium, as shown in [8], will help in developing PL models, which are independent of the antenna properties.

Apart from developing a general useable and a simplified PL model, the aim is also to apply this PL model of a multilayered lossy structure to a heterogeneous human model, where the multilayer structure represents a cross-section of a heterogeneous human body. The PL calculated using the simplified model is compared with the simulated PL in a heterogeneous 3D adult human to verify the applicability in the heterogeneous medium. Thus, instead of using a complete human model for simulations, an equivalent layered structure can be utilized to carry out the PL calculations. Validations are carried out by simulations using comprehensive electromagnetic simulation software, FEKO (EMSS, Stellenbosch, South-Africa), which uses the MoM/FEM (method of moment/finite element method) and SEMCAD-X (SPEAG, Switzerland), which uses the FDTD (finite difference time domain) method.

The path loss occurring from the in-to-out body is critical for link budget calculations, and the PL model can help to find the maximum distance that can be covered between the Tx and the Rx. As it is difficult for the manufacturers to test their system on actual humans, the proposed model can be used by them to evaluate the performance of in-to-out body WBAN systems.

## 2. Configuration

In this section, different configurations are discussed for which the calculations and simulations have been performed.

### 2.1. Lossless to Lossy Medium: Air to Muscle Tissue

**Figure 1.**
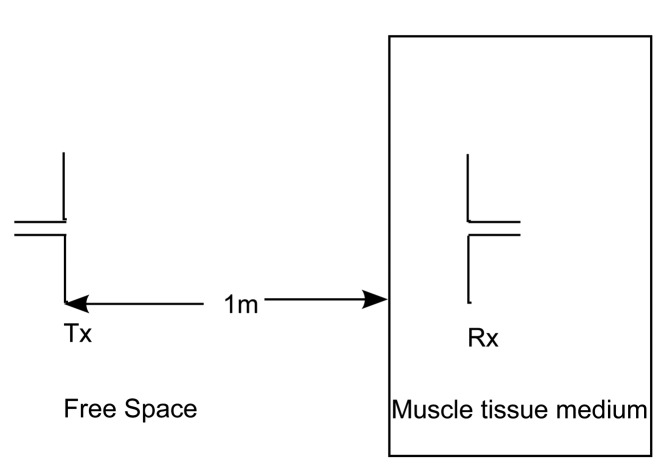
Propagation of the waves from lossless to lossy medium: Air to muscle.

First, a simple two-layer approach is considered here with the Tx placed in a lossless medium, air (*ϵ_r_* = 1 and *σ* = 0 S/m), at a distance of 1 m from the muscle tissue medium, which has a thickness of 0.2 m, as shown in Figure 1. The Tx in air is a free space half-wavelength dipole antenna made of perfect electric conductors (PEC) as designed in [2], and having a length of λ/2 = 0.0612 m, where λ is the wavelength in air at 2.45 GHz. The Rx is an insulated dipole antenna where the dipole arms are PEC surrounded with an insulation made of polytetrafluoroethylene (*ϵ_r_* = 2.07 and *σ* = 0 S/m). The insulation is 2.5 mm in thickness. The Rx is placed in the lossy medium of human muscle tissue. Dipole antennas (length, *ℓ*_1_ = 3.9 cm) are used for this study, as they are the best understood antennas in free space and have a simple structure [7]. The reason for the Tx being placed at 1 m from the muscle tissue layer is so that a plane wave is obtained when the waves reach the muscle tissue layer. We begin the PL modeling using plane waves, hence this requirement. Both of the layers are infinite in extent in the direction perpendicular to the propagation direction.

### 2.2. Lossless to Lossy Medium: Air to Layers Equivalent to an Adult Human Frontal Thorax

**Figure 2.**
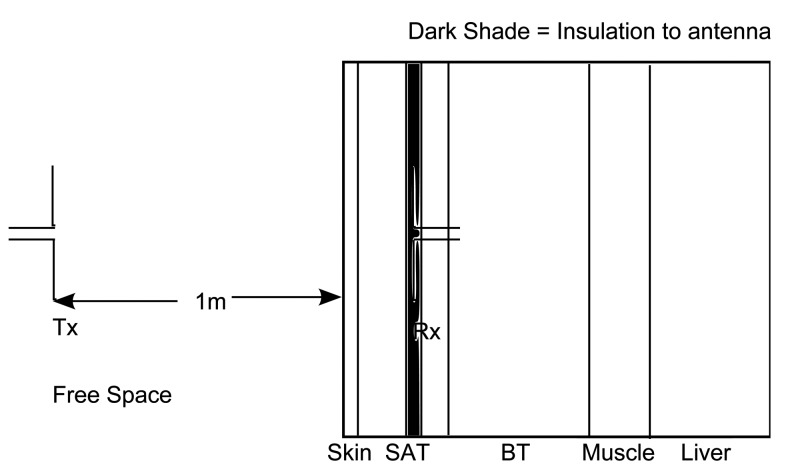
Air to layers equivalent to human frontal thorax.

This configuration consists of a layer of air followed by layers equivalent to the frontal thorax of an adult [16], as shown in Figure 2. The work in [16] provides all possible tissue thicknesses of an adult and child for different body regions and, thus, helps in dividing the human body into various regions of multilayered medium. The Tx dipole antenna is placed in the free space, and the insulated Rx antenna is placed at every 2 mm in the layered model of the frontal thorax of an adult. This layered model consists of the skin (2 mm), subcutaneous adipose tissue (SAT) (23 mm), breast tissue (BT) (30 mm) and muscle tissue (6 mm) and is terminated in the liver (infinite) [16,17]. The Tx antenna is placed at 1 m from the skin. The dielectric parameters of the tissues are shown in Table 1. In this scenario, due to restrictions in modeling using FEKO, the Rx antenna is modeled differently. The insulation of the Rx antenna is modeled as a layer with a thickness of 5 mm in both of the simulators, similar to the other tissue layers, as shown in Figure 2.

### 2.3. Heterogeneous Human Model and Equivalent Tissue Layers

This configuration is for the validation of the simplified PL model. First, the PL is simulated in a heterogeneous human model in any of the regions specified in [16]. This simulated PL is then compared with the PL calculated using the simplified model in a tissue layer equivalent to the heterogeneous human model (in this case, the human frontal thorax).

Active implants are positioned at specific locations inside the human body to carry out special tasks, such as drug delivery, transplanted organ monitoring and functional electrical stimulation. In the case of liver transplantation, implants are placed on the liver for constant monitoring for a period of 7–10 days to report circulation deficiency to physicians [9]. Taking such a scenario into consideration, PL is simulated at various positions of the frontal thorax of Duke, which is an enhanced anatomical model of a 34 year-old adult, from the Virtual Family [18] at 2.45 GHz in SEMCAD-X. Duke has a height of 1.74 m and a weight of 70 kg. The model consists of more than 80 different tissues. The Tx antenna is placed in front of Duke at 1 m, and PL is extracted from the simulations performed in the area of the human frontal thorax. In order to validate the PL, an equivalent tissue layer of the human frontal thorax is considered. PL is calculated in this equivalent tissue layer using the simplified PL model. The equivalent tissue layer consists of skin (2 mm), SAT (5 mm), BT (1 mm), muscle (10 mm) and liver (10 mm) tissue with dielectric properties as shown in Table 1.

## 3. Method

### 3.1. Developing the Simplified PL Model

**Figure 3.**
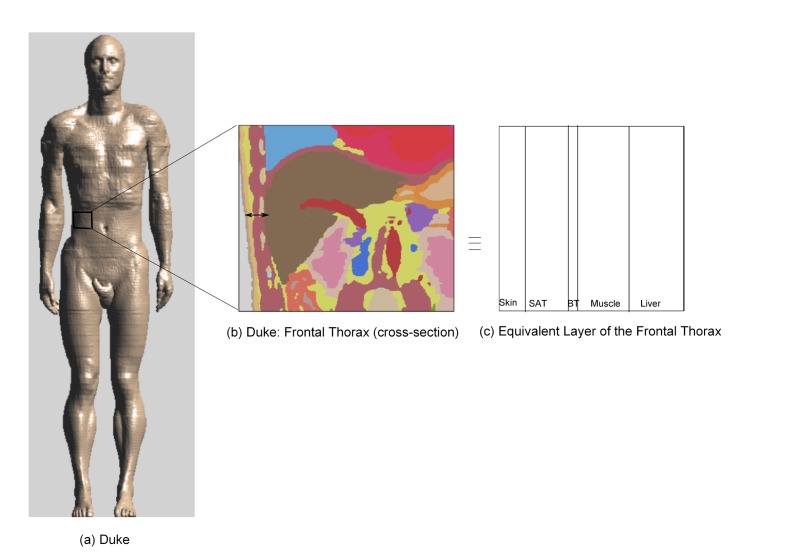
The anatomical adult model: (**a**) Duke, followed by (**b**) the cross-section of the human frontal thorax and (**c**) the equivalent tissue layer of the human frontal thorax.

**Figure 4.**
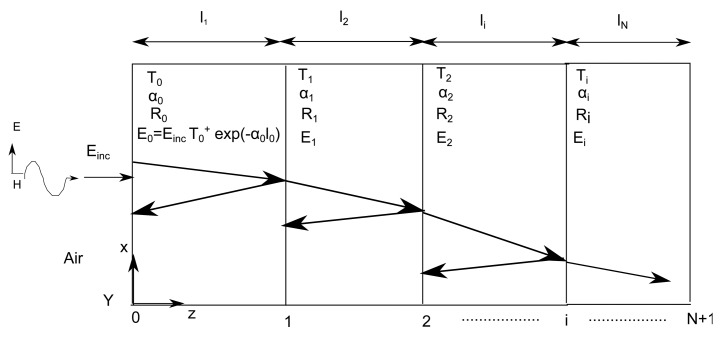
Plane-wave propagation in a multilayer model.

**Figure 5.**
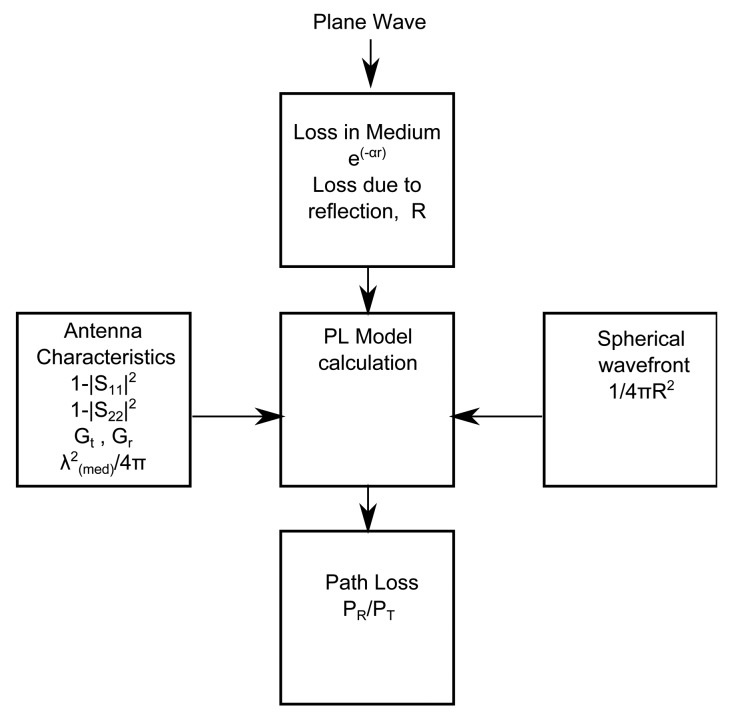
Block diagram of path loss (PL) calculation.

In this section, the steps to develop the simplified PL model are explained. Figure 3 describes our approach towards approximating layered medium in an adult human model. Figure 3b shows the cross-section of the human frontal thorax. Figure 3c is an equivalent layered medium that is used for modeling and is obtained from the highlighted region of Figure 3b. Figure 4 shows a plane wave normally incident to a lossy multilayered medium with N layers of thickness *l_i_*, where i = 1, 2,..,N, and N + 1 interfaces, *T_i_* = transmission coefficient, *R_i_* = reflection coefficient, *α_i_* = attenuation constant of the medium and *η_i_* = characteristic impedance of the medium. The amplitude of the incident plane wave (*E_inc_*) is 1 V/m. As the wave propagates through the medium, it undergoes attenuation and reflections due to the lossy medium and the interface of the layers, respectively. The development of the PL model is explained by means of a flow chart, as shown in Figure 5, and its various blocks are explained as follows.

#### 3.1.1. Loss in the Medium

The attenuation that the plane wave undergoes as it propagates through the multilayered lossy medium is accounted for by *α*, which is the attenuation constant (nepers/meter) of the medium. The time-averaged power per unit area entering each layer is calculated using the Poynting vector, which is given as:

(1) $$P_{i}=\frac{1}{2}\text{Re}(E\times H*),\text{where}\;i=1,2,\ldots ,M$$

The power absorbed in the layers is equal to the difference of the power entering the layer and the power leaving it.

(2) $$P_{i}^{\text{loss}}=P_{i}-P_{i+1},\text{where}\;i=1,2,\ldots ,M$$

#### 3.1.2. Antenna Characteristics

Once the power loss is determined for the plane wave traveling through the layers, the next step is to introduce the antenna characteristics, of the antennas being used, in order to calculate the PL. The antenna characteristics of the two Tx and Rx dipole antennas at 2.45 GHz, as mentioned in Section 2.1 [7], include the reflection coefficients |*S*_11_| and |*S*_22_| of the antennas, respectively. The gains of the Tx and the Rx antennas are *G_t_* and *G_r_*, respectively, and the effective aperture of the Rx antenna is given by $\lambda_{\text{med}}^{2}/(4\pi )$, where λ_med_ is the wavelength of the medium in which the Rx is placed.

#### 3.1.3. Spherical Wavefront

The factor ( $\frac{1}{4\pi R^{2}}$) in (3) takes into account the increase in the spherical wavefront surface [19]. It accounts for the fact that the actual waves are not plane, but spherical, where R is the distance between the Tx and the Rx.

The parameters of the antenna, loss in the medium and the antenna characteristics in a multilayer lossy medium are used in the Friis transmission equation along with the theory of the reflection and transmission of electromagnetic waves for the development of the simplified PL model.

The Friis transmission equation in lossy medium is as follows [19]:

(3) $$\frac{1}{PL}=\frac{P_{r}}{P_{t}}=\frac{\left(1-{\left|S_{11}\right|}^{2})(1-{\left|S_{22}\right|}^{2})\lambda_{\text{med}}^{2}G_{t}G_{r}\exp(-\alpha r\right)}{{\left(4\pi R\right)}^{2}}$$

where (1 − |*S*_11_|^2^) and (1 − |*S*_22_|^2^) are the mismatch efficiency obtained from the reflection coefficients |*S*_11_| and |*S*_22_|. *G_t_* and *G_r_* are calculated depending on their positions, *i.e.*, if they are in free space or in medium [8]. The method of calculating the gain of an antenna in a medium has been described in [8], and the same methodology is applied here.

In the Friis transmission formula, the loss of power in a medium is calculated by *exp*(−*αr*). However, in the case of the propagation of waves in a multilayered lossy medium, reflection and transmission at the interfaces have to be taken into account. Thus, Equation (3) is here modified to:

(4) $$\begin{array}{r} \frac{P_{r}}{P_{t}}=\left(\frac{(1-{\left|S_{11}\right|}^{2})(1-{\left|S_{22}\right|}^{2})\lambda_{\text{med}}^{2}G_{t}G_{r}}{{\left(4\pi R\right)}^{2}}\right)\\ \left(\frac{E_{\text{inc}}^{2}}{2}\overset{n}{\underset{i=1}{\text{∏}}}\frac{T_{i}\;\exp(\alpha_{i})l_{i}}{\eta_{i}}\right)\left(\frac{T_{n}\;\exp(-\alpha_{n}(d-\overset{n-1}{\underset{i=1}{\text{∑}}}l_{i}))}{\eta_{n}}\right)\\ \left(\left(1+R_{n+1}\exp(-2\alpha_{n}\overset{n-1}{\underset{i=1}{\text{∑}}}l_{i}-d\right)\right) \end{array}$$

(5) $$T_{i}=\frac{2\cdot \eta_{i+1}}{\eta_{i}+\eta i+1}$$

(6) $$R_{i}=\frac{\eta_{i}-\eta_{i+1}}{\eta_{i}+\eta_{i+1}},$$

where *T_i_* = transmission coefficient, *R_i_* = reflection coefficient, *α_i_* = attenuation constant of the medium and *η_i_* = characteristic impedance of the medium (see Figure 4).

*E_inc_* is the incident electric field, and, for example, in the first layer, we have, *E*_0_ = *E_inc_ T*_1_ *exp*(*α*_1_*l*_1_), as shown in Figure 4. The Poynting vector is then calculated for all layers, and for the first layer, we obtain, $P_{1}=\frac{E_{1}^{2}}{2\eta_{1}}$, where, *E*_1_ = electric field in Layer 1 and *η*_1_ = characteristic impedance in Layer 1. Substituting all of the parameters in the Friis transmission equation, we obtain a simplified PL model, as shown in Equation (4). This PL model only takes into consideration the transmitted wave in the layered medium, because the medium is lossy, and it has been observed that taking into account the reflected wave does not provide any added value to the PL model due to tissue attenuation and absorption [2]. However, we do retain a reflection coefficient parameter in the last layer, where the antenna is placed, to take into account any changes that might be introduced in case there is high variation of the medium properties between the layer where the antenna is placed and the proceeding layer.

The scattering parameters and the gains of the Tx and the Rx mentioned in Tables 2 and 3 are then used for the PL calculation. In Table 3, *S*_22_*__Med__* [dB] is the reflection coefficient and *G_r_Med__* is the gain of the insulated dipole antenna in the medium and is calculated using the method from [8].

Thus, to summarize, we start developing a PL model by using a plane wave in a layered medium and calculating the Poynting vector, then the antenna characteristics are introduced in the Friis transmission formula to develop a PL model for layered tissues to calculate the PL in it.

### 3.2. Simulation Settings

In order to validate the PL model, simulations are carried out for various configurations (Section 2) in FEKO and SEMCAD-X. For accurate modeling in FEKO, segmentation rules are adhered to (segment length = λ*_res_*/12, edge length = λ*_res_*/12). The source used is a current source. SEMCAD-X enables a non-uniform gridding. The maximum grid step in the muscle tissue medium is 1 mm at 2.45 GHz. The source used is a voltage source. The SEMCAD-X and the FEKO simulations both make use of a half-wavelength dipole in free space and an insulated dipole in the lossy medium (Section 3.1). These simulations calculate the PL and the results are compared to the PL model.

## 4. Results: Path Loss

### 4.1. Lossless to Lossy Medium: Air to Muscle

**Figure 6.**
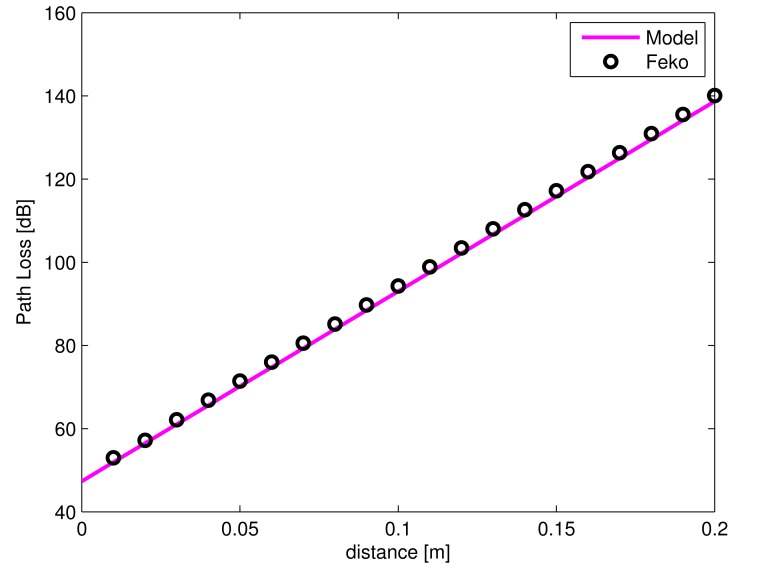
Simulated and modeled PL of the insulated dipole in an air-muscle configuration *versus* the separation distance between antennas.

In this section, the results of the configuration from Section 2.1 are discussed, which consists of a simple two-layer model of free space and muscle tissue with the Tx and Rx dipole antennas at 2.45 GHz. Figure 6 shows the interface between free space and muscle tissue at 0 m. It can be seen that PL increases with increasing distance *d*, where *d* is the separation between the Tx placed in free space (at 1 m from the muscle tissue layer) and the Rx placed in the muscle medium. A very good agreement is observed between the PL model and the simulations using the MoM (FEKO) in the muscle tissue medium with a maximum deviation of only 1.4 dB and an average deviation of 1 dB. Thus, the PL model can be used instead of carrying out simulations for a two-layered model.

### 4.2. Calculated PL versus Simulated PL in Layered Tissues Equivalent to the Adult Frontal Thorax

**Figure 7.**
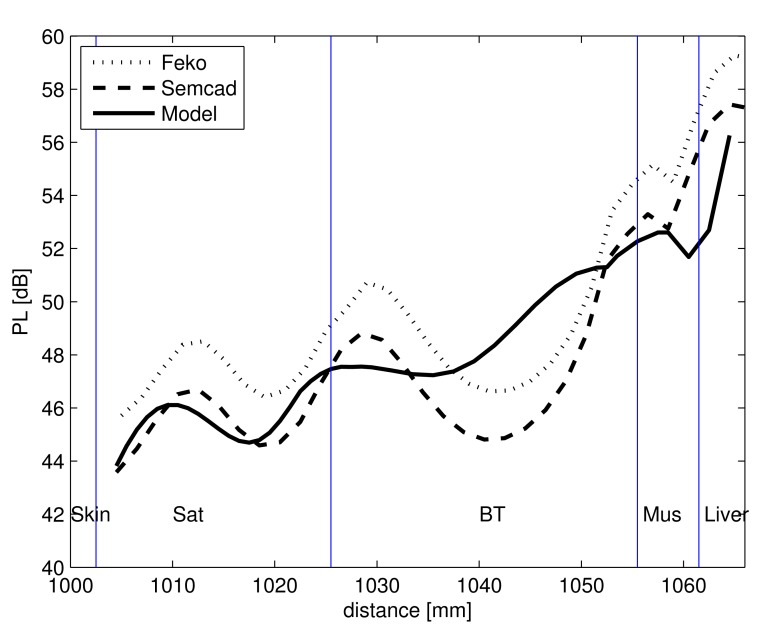
Simulated (FEKO and SEMCAD-X) and PL model of the insulated dipole in multilayer lossy medium *versus* the separation distance between antennas.

The results obtained from the configuration mentioned in Section 2.2 are discussed here. Figure 7 shows the PL in the lossy layered medium obtained by calculations and MoM (FEKO) and FDTD (SEMCAD-X) simulations. The simulations, as well as the calculations from the simplified PL model display a standing wave pattern in the layered medium. The standing wave behavior is caused by the reflections of the propagating waves at different tissue layer interfaces [16]. It is noted that for this configuration, also the interface of the insulation of the receiving antenna is responsible for the wave pattern seen in Figure 7. Since the dielectric properties of the antenna insulation (*ϵ_r_* = 2.07 and *σ* = 0 S/m) are very different from that of the lossy human tissue layers, it causes a pronounced standing wave pattern. In spite of developing a 1D simplified PL model and comparing it with a 3D-model, the PL model shows very good agreement with the simulations and shows a maximum deviation of 3.3 dB and 2.8 dB and an average deviation of 2.9 dB and 2.5 dB with that of the SEMCAD-X and the FEKO simulations, respectively.

For simulations carried out in the layered medium, the time required for a sample size of 30 antenna separations is 22,320-times more than that required for calculation using the PL model, thus saving valuable time (for a system with a Windows 7, 64-bit operating system, with an Intel CPU at 2.50 GHz and 64 GB of RAM). There is a deviation of 2 dB between the two simulations in Figure 7, and this can be attributed to the way the antennas and the layered medium are modeled. In FEKO, the length of the layers are modeled as infinite medium, while that in SEMCAD-X is finite and equal to 40 cm.

Next, the applicability of the PL model in the heterogeneous human body is verified.

### 4.3. Validation: 3D Heterogeneous Human Frontal Thorax Model vs. Equivalent Tissue Layer

This section is based on the configuration explained in Section 2.3 and is used for carrying out the simulations in a 3D heterogeneous adult model. The results from these simulations are validated by calculating the PL in tissue layers that are equivalent to the human frontal thorax using the simplified PL model. The thicknesses and the dielectric properties of the tissue layers are as mentioned in Section 2.3.

**Figure 8.**
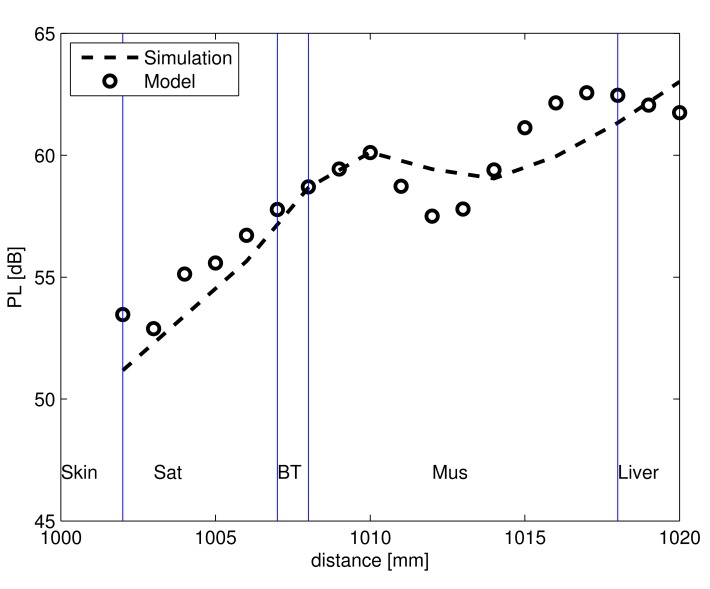
Simulated PL in the heterogeneous human Duke and PL model *versus* the separation distance between antennas.

Figure 8 shows the comparison of the calculated PL model in equivalent tissue layers and the simulated PL in Duke *versus* the distance between the Tx and the Rx antenna. In the heterogeneous human body, the receiving antenna moves through the various layers, and PL is obtained. The layers in the frontal thorax of Duke for skin, SAT and breast tissue are lesser in thickness (2 mm, 5 mm and 1 mm) compared to the thickness of the insulated antenna (5 mm). Thus, when the antenna is placed in a specific layer, the layers that surround the antenna are not similar to the layer in which it is placed. Thus, a deviation can be observed between the simulated and calculated PL and also a change in the standing wave pattern between the calculations and the simulations. The standing wave pattern observed in the Figure 8 is due to the reflections caused by the insulation of the antenna. There is a maximum deviation of 1.9 dB and an average deviation of 1.7 dB between the calculated PL model of Equation (4) and the PL from simulation.

For FDTD simulations carried out in Duke, the time required for a sample size of 30 antenna separations is 86,400-times more than that required for calculation using the PL model, *i.e.*, it takes approximately five days to carry out the simulations, while the calculations using the simplified PL model takes only five seconds.

## 5. Link Budget for Wireless Communication

**Figure 9.**
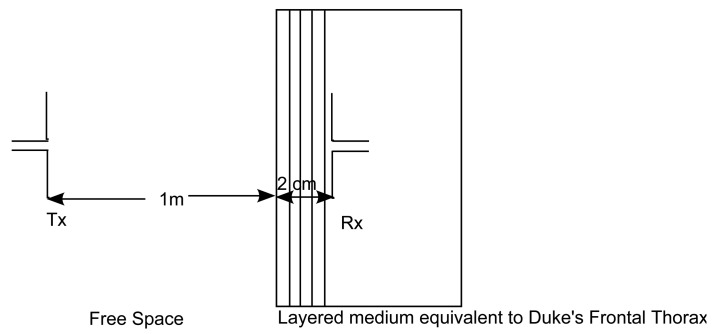
Configuration to calculate the link budget for the in-to-out body scenario.

We develop a link budget for an out-to-in body scenario to check for communication between the Tx placed in free space and the Rx placed in the human body Such a scenario is important in a hospital where a patient with an on- or in-body implant can transmit/receive data to/from a device placed outside the body For carrying out the link budget calculations, the Tx is placed at a distance of 1 m from the the human body, and the Rx is placed at a distance of 20 mm in the layered medium equivalent to the frontal thorax region of Duke (example taken from Section 2.3; refer to Figure 9). Our goal here is to check if communication is possible between the Tx and Rx when they are separated by a distance of 102 cm. The parameters selected for developing the link budget are listed in Table 4. The link budget is developed at a frequency of 2.45 GHz for an input power of 25 *μ*W (from the European Research Council (ERC) limitation [5]). If the carrier *C* to noise *N*_0_ ratio, *C*/*N*_0_ of the link between the Tx and the Rx exceeds the required *C*/*N*_0_, then wireless communication is possible between the Tx and the Rx.

(7) $$\text{Link}\;C/N_{0}=P_{t}-L_{tx\;\text{feed}}+G_{t}-L_{t}-L_{rx\;\text{feed}}-N_{0}$$

(8) $$\text{Required}\;C/N_{0}=(E_{b}/N_{0})+10\log_{10}(B_{r})-G_{c}+G_{d}$$

(9) $$L_{f}=10\log_{10}{\left(\frac{4\pi d}{\lambda }\right)}^{2}(dB)$$

(10) $$N_{0}=10\log_{10}(k)+10\log_{10}(T_{i})(dB/Hz)$$

(11) $$T_{i}=T_{0}(NF-1)(K)$$

By the use of Equations (7) and (8), the link *C*/*N*_0_ and required *C*/*N*_0_ can be calculated, respectively (for the notations, see Table 4). *PL_Model_* takes into account path loss, calculated by the simplified model, occurring in the body, and it also consists of the antenna gain; thus, the *G_r_* is not added separately in Equation (7). *PL_Sim_* is the path loss from the FDTD simulation. The link budget calculation takes the example of the scenario of the human frontal thorax discussed in Section 4.3. From the Table 4, it is calculated that the link *C*/*N*_0_ exceeds the required *C*/*N*_0_, for a distance of 102 cm (a distance of 1 m between the Tx and the medium and 2 cm between the start of the medium and the Rx) in the case of both the model, as well as the simulation; thus, wireless communication is possible.

## 6. Conclusions

We developed for the first time a simple, fast and efficient antenna-independent PL model for in-out body communication for multilayered lossy tissues and heterogeneous human medium at 2.45 GHz, eliminating the need for time-consuming simulations. A maximum deviation of only 1.9 dB and an average deviation of 1.7 dB is observed between the PL model and the simulated PL in the heterogeneous medium, and the time taken to carry out the simulations is 86,400-times greater than that required for calculation for a sample size of 30 antenna separations. The simplified PL model can be used to design any in-out body communication systems and also to determine the link budget for such systems. As an application, the proposed model is used to calculate the range of distances between the Tx and the Rx in the case of various scenarios, such as in a hospital environment where the data transfer takes place from in-, on-, or off-body devices to a receiving unit. For the future extension of this work, we would like to develop the PL model for the MICS (medical implant communication service) band at a frequency of 402–405 MHz for a different set of antenna designs. We would also like to use this PL model for the prediction of SAR (specific absorption rate) in the layers.

## Figures and Tables

**Table 1.** Values of *ϵ_r_* and *σ* [*S*/*m*].

**Parameter**	***Skin***	***SAT***	***Breast Tissue***	***Muscle***	***Liver***
*ϵ_r_*	38	10.8	5.15	52.7	43
*σ* [*S*/*m*]	1.46	0.27	0.14	1.74	1.69

**Table 2.** Parameter values of the Friis transmission formula.

**Parameter**	***S*_11_ (*dB*)**	***S*_22_ (*dB*)**	***G****_t_* **(*dBi*)**	***G****_r_* **(*dBi*)**
Muscle tissue	−11.57	−11.57	7.5	7.5
Air to Muscle	−10.45	−11.57	2.14	7.5
Air to Layers	−10.45	*S*_22_*__Med__*(*See* Table 3)	0.11	*G_r_Med__* (*See* Table 3)

**Table 3.** Values of *S*_22_*__Med__* (*dB*) and *G_r_Med__* (*dBi*).

**Parameter**	***Skin***	***SAT***	***BreastTissue***	***Muscle***	***Liver***
*S*_22_*__Med__*	−10.71	−8.52	−8.76	−11.57	−10.95
*G_r_Med__*	10.47	5.61	3.82	11.46	11.04

**Table 4.** Parameter values: Link budget calculation for a distance of 102 cm of separation between the Tx and the Rx.

**Tx**		**Rx**	

Frequency (MHz)	2450	Rx antenna gain Gr (dBi)	11.04
Tx power *P_t_* (*μ*W)	25	Feeding Loss *L_rx feed_* (dB)	0
Tx power *P_t_*(dBm)	−16	Ambient Temperature T (K)	310.0
Feeding Loss *L_tx feed_* (dB)	0	Receiver NF (dB)	3.5
Tx antenna gain *G_t_*	2.14	Boltzmann constant *k*	1.38 × 10^−23^
		Noise power density *N*_0_(dBm/Hz)	−199.70

**Signal Quality**		**Propagation**	

Bit error rate	1.0 × 10^−5^	Free space loss *L_f_*(dB)	52.02
Bit rate *B_r_* (*Mb*/*s*)	2	*PL_Model_* (dB)	62
Eb/N0 (ideal PSK) (dB)	9.6	*PL_Sim_* (dB)	63
Coding gain *G_c_*	0	Total propagation loss *L_t_*(dB) = PL	
Fixing deterioration *G_d_* (dB)	2.5	where PL = *PL_Model_* for Model and = *PL_Sim_* for Simulation	

Distance = 2 cm in the Tissue Layer (Tx placed at 1 m from tissue)		Total distance = 102 cm	
Link (PL Model) *C*/*N*_0_ (dB/Hz)	123.84	Link (PL Simulation) *C*/*N*_0_ (dB/Hz)	122.84
Required *C*/*N*_0_ (dB/Hz)	75.11		
